# 

**Published:** 1917-03-24

**Authors:** 


SUPPLEMENT TO "THE HOSPITAL," May 19, 1917.

WAA

### THE HOSPITAL

*THE MODERN NEWSPAPER OF ADMINISTRATIVE MEDICINE AND INSTITUTIONAL LIFE*

INCLUDING

### ADMINISTRATION, NATIONAL INSURANCE, HEALTH
AND
### THE INTERESTS OF ALL INSTITUTIONAL WORKERS

OCTOBER 1916 TO MARCH 1917

VOLUME LXI

LONDON

PUBLISHED BY THE SCIENTIFIC PRESS (LIMITED), AT *THE HOSPITAL* BUILDING

28 & 29 SOUTHAMPTON STREET, STRAND, W.C.

MDCCCCXVII

### THE STETHODYOSCOPE.

It seems to be particularly true of the inventing of surgical instruments that there is nothing new under the sun, as witness recent correspondence about the pneumatic tourniquet. Recently a stethoscope with one chest-piece, but two sets of ear-fitments, has been devised for purposes of instruction, so that professor and pupil can auscult simultaneously, a plan which is obviously to the benefit of the latter. A somewhat similar procedure has, of course, been devised in laryngoscopy, but we do not remember having seen the "sociable" stethoscope before advocated. It should be of real service.

### HOSPITAL AND INSTITUTIONAL RESULTS.

**Northampton General Hospital.**—This is another of the comparatively small group of hospitals which make up their accounts for odd periods, and thereby deny themselves the useful privilege of being able to compare their returns with those of other institutions. In the year ending July 31, 1916, the total ordinary income was £22,988, including £8,743 paid on account of military and private patients. The extraordinary income was £3,714, and the total expenditure £22,086.

**Aberdeen Royal Infirmary.**—This is one of the first reports, containing a full record of the work for 1916, that we have received. There was again a deficit on the accounts, income being £14,464 and expenditure £17,413. The cost of provisions has increased by £1,340.

**St. Andrew's Hospital, Dollis Hill.**—One of the first complete reports for 1916. The hospital was founded in 1913, and is chiefly intended for middle-class people, either as free or paying patients. During the past year a number of wounded and sick soldiers and refugees have been received. Total income, £7,155, including £4,656 patients' payments; expenditure, £7,224. There has been a small deficit for four years in succession.

**Aberdeen Royal Asylum.**—Another complete report for 1916, with the accounts made up to December 31. The income was £42,759, including £39,741 for the board of mentally deficient patients. The expenditure was £42,524. The total number of cases on the register at the end of the year was 887.

**St. Mary's Free Hospital for Children, New York.**—This report is for the twelve months ending September 30, 1916, of a hospital established in the year 1870 by the Sisters of St. Mary for the medical and surgical care of sick, maimed, and crippled children. The hospital in New York has accommodation for 126 children; the Branch Summer Hospital, in Norwalk, can take eighty more. There is also a convalescent home for twenty patients, and a dispensary for out-patients. The annual cost of the whole work is about £13,600, and there is an endowment fund with an income of £4,600.

**St. Luke's Hospital, New York.**—The report of this general hospital is for the year ending September 1916, during which period 7,328 patients were treated in the wards and private rooms. The income was £78,745; the expenditure was £80,385. The report includes a complete list of members of the board of management and medical officers since 1850, and the names of graduates of the training-school for nurses, with particulars of their present occupations, since 1890.

**King Edward VII. Sanatorium, Midhurst.**—This report is for the period from July 1915 to July 1916. A table of ultimate results shows the after-history of cases after leaving the sanatorium; for instance, of 125 undeniable cases of tuberculosis discharged in 1906-7, seventy-two were known to be dead in 1916. The report is entirely medical in character—no accounts are presented.

**Royal Southern Hospital, Liverpool.**—The full accounts for 1916 are contained in the report. The total income for the year was £15,276, including legacies £1,886, and the expenditure £19,241. There is a debt to the bankers of £9,317. A new department for the treatment of tubercular conditions and some other cases of bacterial origin by ultra-violet rays has been found very useful.

**Royal Maternity Charity of London.**—621 cases were attended in 1916 and 641 infants born, all but eight surviving. Income £1,237. Expenditure £1,429. The report consists of four pages.

**Pontypool and District Hospital.**—One of the earliest full reports. Total income £4,025, total expenditure, including £777 transferred to building account, £3,620. Individual subscriptions, workmen's collections, etc., are detailed in the actual income and expenditure account.

### KING EDWARD'S HOSPITAL FUND FOR LONDON.

We are asked to state that hospitals in the County of London, or within nine miles of Charing Cross, desiring to participate in the grants made by this Fund for the year 1917 must make application before the 31st instant to the Hon. Secretaries, 7 Walbrook, E.C.4. Applications will also be considered from convalescent homes which are situated within the above boundaries, or which, being situated outside, take a large proportion of patients from London. Applications will also be considered from sanatoria for consumption which take patients from London, or which are prepared to place beds at the disposal of the Fund for the use of patients from London hospitals.

### Matrons' and Sisters' Appointments.

Sandon Hall, Weston, Stafford.—Miss E. Sharman has been appointed night sister. She was trained at the Great Northern Central Hospital, and has since been sister in charge of an officers' hospital in London.

Workhouse Infirmary, Caerleon, near Newport, Mon.—Miss E. M. O'Connor has been appointed night sister and assistant superintendent nurse. She was trained at Chorlton Union Infirmary, Manchester.

St. John's Hospital for Diseases of the Skin.—Miss F. I. Yule has been appointed sister. She was trained at Woolwich Infirmary, Plumstead, where she later took sister's duties.

Perth Royal Infirmary.—Miss J. W. Wightman has been appointed sister. She has been ward and theatre sister at the Royal Bucks Hospital, Aylesbury.

Arnold Auxiliary Hospital, Doncaster.—Sister Rose Kendal has been appointed sister. She trained at the Salisbury General Infirmary, and has been sister-in-charge at a military hospital in Bedfordshire and at the Royal Naval Hospital, Truro.

Military Hospital, Cambridge Road, Bethnal Green, N.E.—Miss Isabella K. C. Cochrane has been appointed sister. She was trained at St. Bartholomew's Hospital, after which she went to Australia, and was sister at the Children's Hospital, Brisbane, and co-matron of the Clair Private Hospital, Brisbane.

Newcastle-on-Tyne Poor-Law Infirmary.—Miss M. E. Watson has been appointed ward sister. She was trained at the Poplar and Stepney Sick Asylum, and has since been theatre and casualty ward sister at the Cottage Hospital, South Elmsall, Doncaster.

Western Fever Hospital, Fulham.—Miss Grace Ellis has been appointed sister. She was trained at the City of Westminster Infirmary, Hendon, and has since been sister at the Holborn Infirmary, Archway Road.

Ham Green Sanatorium, Bristol.—Miss E. M. Doyle, Miss Alice Hill, and Miss Grace Kerr have been appointed sisters. Miss Doyle was trained at Lewisham Infirmary, and has since been night sister at Wimbledon Borough Hospital and sister at Dover Borough Hospital. Miss Hill was trained at Swansea General Hospital and at the Eastern Fever Hospital, Homerton. She later became sister at the South Eastern Hospital, New Cross, and at the City Sanatorium, Yardley Road, Birmingham. Miss Kerr was trained at Bolton Infirmary and at the Ham Green Sanatorium.

**Note.—Particulars of Appointments for insertion in this column will be welcomed.**

### Annual Meetings.

Charing Cross Hospital, Agar Street, Strand, W.C.—Friday, March 23, at 5 p.m., in the Council Room of the hospital, Mr. George Verity, J.P., the Chairman of the hospital, presiding.

National Hospital for the Relief and Cure of the Paralysed and Epileptic (Albany Memorial), Queen Square, W.C.—Tuesday, March 27, 1917, at 4.30 p.m. at the hospital.

Newsvendors' Benevolent and Provident Institution.—Tuesday, March 27, in the Board Room, Memorial Hall, Farringdon Street, London, at 6.30 p.m. precisely, Colonel Lord Burnham, M.A., J.P., the President, in the chair.

Italian Hospital, Queen Square, W. C.— Thursday, March 29, 1917, at 4.30 p.m., His Excellency the Italian Ambassador, President of the hospital, in the chair.

**Rates of Subscription:** Twelve months, 6/6; Six months, 4/-; Three months, 2/-; post free to any address in the United Kingdom. Abroad, Twelve months, 8/8; Six months, 5/-; Three months, 2/6, post free. THE HOSPITAL "Index" to Volume LX., April to September 1916, is now ready, price 2½d. per copy, post free. Address The Manager, "THE HOSPITAL," The Hospital Building, 28/29 Southampton Street, Strand, London, W.C. 2.

